# Loculated Fluid Visualized in Hepatorenal Space with Point-of-care Ultrasound in Patient with Pelvic Inflammatory Disease Caused by Group A Streptococcus: Case Report

**DOI:** 10.5811/cpcem.6663

**Published:** 2024-08-09

**Authors:** Neil Makhijani, Samuel E. Sondheim, Turandot Saul, Elizabeth Yetter

**Affiliations:** Mount Sinai Morningside-Mount Sinai West, Icahn School of Medicine at Mount Sinai, Department of Emergency Medicine, New York, New York

**Keywords:** point-of-care ultrasound, pelvic inflammatory disease, peritonitis, case report

## Abstract

**Introduction:**

Point-of-care ultrasound (POCUS) is a screening and diagnostic modality frequently used in the emergency department to assess patients with abdominal pain.

**Case Report:**

We present a case describing the unusual finding of intraperitoneal fluid with loculations visualized in the right upper quadrant of the abdomen in a patient ultimately diagnosed with pelvic inflammatory disease (PID) with ruptured tubo-ovarian abscess caused by group A streptococcus (GAS), a pathogen rarely implicated in the disease.

**Conclusion:**

Uncommon findings on abdominal POCUS should trigger further investigation. In a patient not responding to antibiotics administered for typical PID coverage, GAS should be considered as a possible etiology and a penicillin-based antibiotic administered to prevent progression to tubo-ovarian-abscess formation, peritonitis, and sepsis.

## INTRODUCTION

Pelvic inflammatory disease (PID) is an infection of the upper genitourinary tract typically seen in sexually active women. While some patients are asymptomatic, signs and symptoms may include abdominal pain, dyspareunia, cervical motion tenderness, and vaginal discharge. Pelvic inflammatory disease is most often caused by *Neisseria gonorrhoeae, Chlamydia trachomatis*, or other colonizing flora of the genitourinary tract.^1^ National Health and Nutrition Examination Survey data from 2013–2014 revealed that 4.4% of women between the ages of 18–44 years who were sexually active reported a past medical history of a PID diagnosis.^2^ Infertility and tubo-ovarian abscesses (TOA) are known complications of PID.

Point-of-care ultrasound (POCUS) is a screening and diagnostic modality frequently used in the emergency department (ED) to assess patients with abdominal pain. In this case report we describe the unusual finding of intraperitoneal fluid with loculations visualized in the right upper quadrant of the abdomen. This finding, when coupled with the clinical history and physical examination, led to additional diagnostic imaging and ultimately a diagnosis of a ruptured TOA. Grossly purulent fluid was discovered during peritoneal wash out in the operating room. Culture of the peritoneal fluid grew group A streptococcus (GAS), a pathogen rarely implicated in PID. We found no other reported cases of PID caused by GAS with associated POCUS findings of septated or loculated intraperitoneal fluid in the hepatorenal space in a review of the medical literature.

## CASE REPORT

A24-year-old female G1P0010 with a past medical history of a right ovarian cyst and a medical termination of pregnancy three months prior presented to the ED with sudden onset of abdominal pain that woke her from sleep. She reported the pain as severe and diffuse, notably worse in the upper abdomen, with associated nausea and lower back pain. Additionally, the patient reported white vaginal discharge. She denied any fever, chills, vomiting, chest pain, shortness of breath, diarrhea, or constipation.

On physical examination, the patient had an oral temperature of 99° Fahrenheit (37.2° Celsius), tachycardia of 108 beats per minute, blood pressure of 106/71 millimeters of mercury, 97% oxygen saturation on room air, and a diffusely tender abdomen with voluntary guarding and rebound tenderness suggestive of peritonitis. A POCUS was performed to assess for intraperitoneal free fluid. It revealed a complex collection of fluid with internal echoes in the hepatorenal space raising concern for a loculated infection (See Image and Video).

Bowel gas obscured the transabdominal suprapubic images of the pelvic organs. Intravenous (IV) morphine was administered for pain control. Pelvic examination revealed cervical motion tenderness and white discharge coming from the external cervical os. Laboratory analysis was remarkable for a white blood cell count of 18.9 × 10^3^ per microliter (K/μL) (reference range 4.5–11.0 K/μL) with a neutrophil predominance of 93%. Electrolytes, lipase, hepatic function panel, and lactate were within normal limits. Serum β-human chorionic gonadotropin was negative, and there was an unremarkable urinalysis. Broad spectrum antibiotics were initiated.

**Population Health Research Capsule**

What do we already know about this issue?

*Point-of-care ultrasound (POCUS) is a readily available tool to assist in differentiating abdominal pain.*

What makes this presentation of disease reportable?

*Unusual POCUS findings led to further workup revealing a ruptured tubo-ovarian abscess (TOA) that ultimately grew group A streptococcus (GAS) despite no expected risk factors.*

What was the major learning point?

*Unusual POCUS findings should trigger further investigation; consider GAS as an etiology in patients who do not respond to typical pelvic inflammatory disease (PID) coverage.*

How does this improve emergency medicine practice?

*In PID/TOA patients who have been appropriately treated but continue to have infectious symptoms and unusual POCUS findings, consider GAS.*

Transvaginal ultrasound performed by radiology reported asymmetric enlargement of the right ovary with a complex, 4.2-centimeter (cm) cyst. There was asymmetric increased flow to the right ovary on Doppler evaluation. Gynecologic consultation was obtained, and due to concern for right ovarian torsion/detorsion the patient was taken for diagnostic laparoscopy. The patient was found to have a ruptured TOA with 200 milliliters (mL) of purulent fluid in the peritoneal space. A peritoneal washout and cystectomy were performed, and the patient was admitted for IV ceftriaxone, doxycycline, and metronidazole. She was discharged on postoperative day three with a 14-day course of oral doxycycline and metronidazole. The intraperitoneal fluid sample collected during laparoscopy grew GAS.

The patient returned one month later with fever, chills, yellow vaginal discharge, and lower abdominal pain. Contrast-enhanced computed tomography confirmed a recurrent TOA, which was treated with IV piperacillin-tazobactam and clindamycin per recommendation by the infectious disease consultant. The infection responded well to antibiotics, and the patient was discharged without further recurrence to date.

## DISCUSSION

Abdomino-pelvic POCUS may guide triage, diagnosis, and management, assisting the clinician in investigating a range of disease entities including biliary pathology, abdominal aortic aneurysm, or TOA.^3^–^5^ Bedside ultrasound is generally readily available in the ED setting and may serve as an additional modality to identify unusual findings early in the patient’s clinical course. It does not require ionizing radiation and is not time intensive to perform. Clinicians may consider early use of POCUS for peritoneal findings, as evidenced in this case presentation. In conjunction with information obtained on history and physical examination, the unusual, right upper quadrant POCUS findings of intraperitoneal fluid with loculations caused concern for a disseminated pelvic infection. This was confirmed as a ruptured TOA with a moderate amount of purulent, intraperitoneal free fluid on the operative report. Fitz-Hugh-Curtis syndrome was a concern given the complicated fluid visualized with POCUS, but there was no comment of violin-string adhesions, adhesions, or fibrous adhesions between the anterior hepatic capsule and parietal peritoneum noted on the laparoscopic operative report, although it is unknown whether this anatomic area was evaluated.

Intraperitoneal fluid is easily visualized on ultrasound, and when complex fluid with loculations is encountered, the differential diagnosis includes malignancy, inflammation, and infectious processes.^6^ Similar findings of septated intraperitoneal fluid have been documented in cases of *C trachomatis* in PID,^7^ cholecystitis,^8^ and tubercular peritonitis.^9^

In addition to the uncommon POCUS examination findings, fluid cultured from the abdominal cavity grew GAS. Outside of peripartum infections, those associated with an intrauterine device (IUD) and other invasive gynecologic procedures, GAS infections are rare. This patient reported no recent gynecologic instrumentation. Snyder and Schmalzle described a case of a patient in septic shock from PID, where GAS was the causative organism.^10^ They then conducted a literature review of 13 other cases of PID due to GAS infection. All but one of the cases reported abdominal pain, and 8/13 reported genitourinary symptoms such as vaginal discharge or gastrointestinal symptoms such as nausea, vomiting, and/or diarrhea.

This patient’s clinical course was complicated by a recurrent TOA and sepsis on a return ED visit after initially responding to standard PID antibiotic coverage. She ultimately required penicillin to treat GAS. While both PID and GAS are easily treated by antibiotics, if left untreated they may progress to septic shock and death. In patients diagnosed with PID but not improving on standard antibiotic regimens, clinicians should consider other causes such as GAS and streptococcal toxic shock syndrome.

## CONCLUSION

Group A streptococcus is a rare cause of pelvic inflammatory disease, usually seen in the peripartum period, in patients with an IUD or who have had other recent, invasive gynecologic procedures. In a patient not responding to antibiotics administered for typical PID coverage, GAS should be considered as a possible etiology and a penicillin-based antibiotic administered to prevent progression to tubo-ovarian abscess formation, peritonitis, and sepsis, particularly in cases of returning patients previously treated with antibiotics. Bedside ultrasound should be performed in patients presenting with abdominal pain, and uncommon findings should trigger further investigation. To our knowledge, no other cases of loculated fluid in the hepatorenal space associated with PID have been reported.

## Supplementary Information

Video. Right upper quadrant ultrasonography using curvilinear probe fanning through the coronal plane demonstrating ascites and septations in a patient ultimately diagnosed with pelvic inflammatory disease.

## Figures and Tables

**Image:**
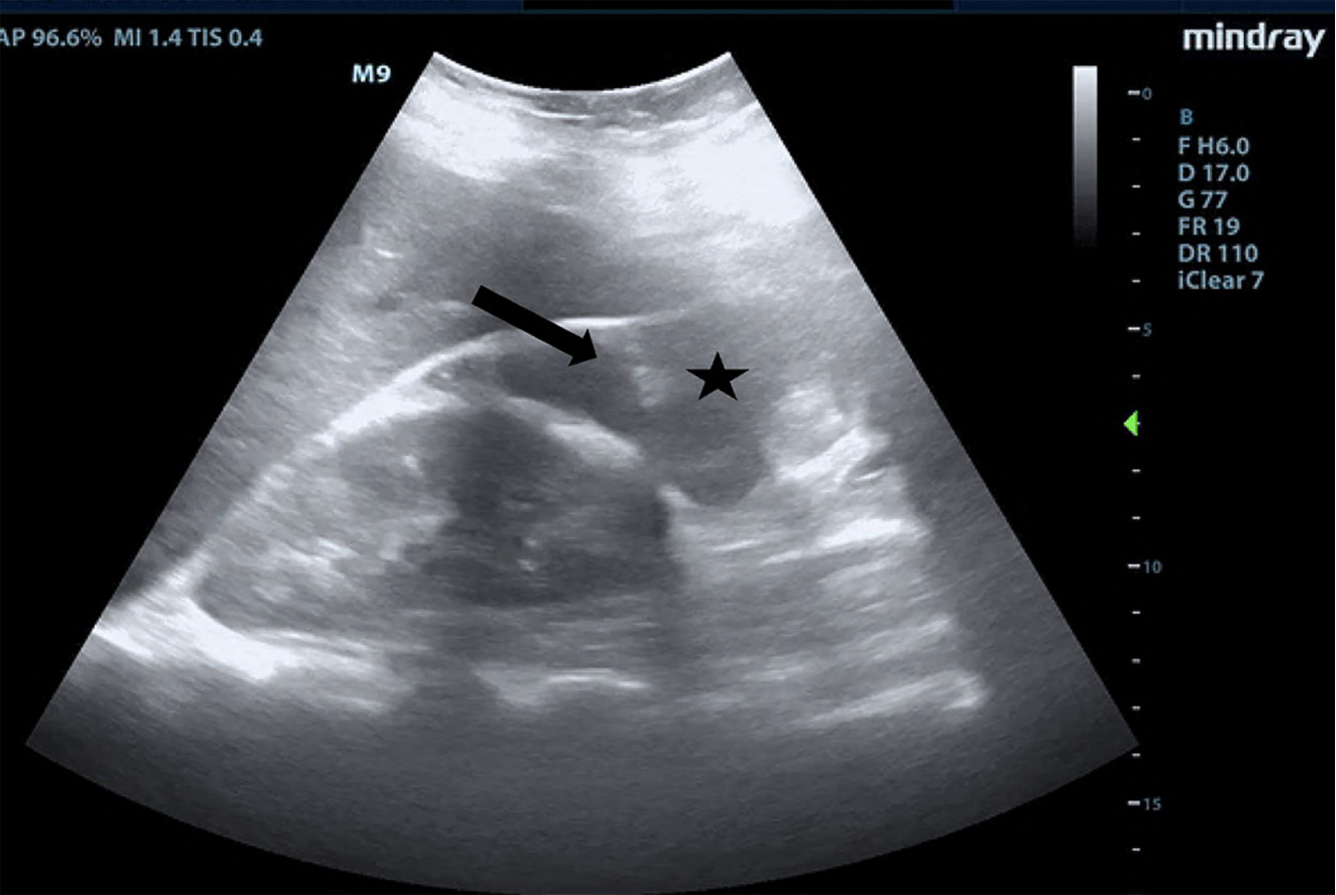
Right upper quadrant ultrasonography using curvilinear probe in the coronal plane demonstrating ascites (black star) and septations (black arrow) in a patient ultimately diagnosed with pelvic inflammatory disease.
